# Is there a bidirectional association between sedentary behaviour and cognitive decline in older adults? Findings from the Irish Longitudinal Study on Ageing

**DOI:** 10.1016/j.pmedr.2021.101423

**Published:** 2021-07-01

**Authors:** Carlijn M. Maasakkers, Jurgen A.H.R. Claassen, Siobhan Scarlett, Dick H.J. Thijssen, Rose Anne Kenny, Joanne Feeney, René J.F. Melis

**Affiliations:** a Department of Geriatrics/Radboud Alzheimer Center, Radboud Institute for Health Sciences, Radboud University Medical Center, Nijmegen, the Netherlands; b The Irish Longitudinal Study on Ageing, Trinity College Dublin, Dublin, Ireland; c Department of Geriatrics/Radboud Alzheimer Center, Donders Institute for Brain, Cognition and Behavior, Radboud University Medical Center, Nijmegen, the Netherlands; d Department of Physiology, Radboud Institute for Health Sciences, Radboud University Medical Center, Nijmegen, the Netherlands; e Research Institute for Sport and Exercise Science, Liverpool John Moores University, Liverpool, United Kingdom; f Mercer’s Institute for Successful Ageing, Department of Medical Gerontology, St James’s Hospital, Dublin, Ireland

**Keywords:** Sedentary behaviour, Cognitive decline, Sitting, Dementia, Older adults

## Abstract

Research on whether sedentary behaviour (SB) is related to cognitive decline in older individuals is conflicting, potentially caused by methodological differences in previous studies. To inform public health policies, we analysed both the forward and reverse association across four-years between subjective TV time and objectively-measured SB and four cognitive outcome measures in older adults. The Irish Longitudinal Study on Ageing (TILDA) quantified time spent watching TV using a questionnaire and objective physical activity patterns with a GENEActiv accelerometer. Mixed model analysis examined whether these two measures of SB related to changes in cognitive function (immediate and delayed recall, MMSE, and animal naming task) during a four-year follow-up period. Furthermore, the reverse association between changes in cognition over the preceding four years and SB was investigated. We included 1,276 participants (67 ± 9 years). Longitudinally, every hour of objective SB per day was associated with a −0.01 (95%CI = −0.03;−0.00) lower MMSE score per year. Reversely, a worse decline in immediate and delayed recall over the preceding waves was related to slightly more objective SB (B = −0.24 (95%CI = −0.41;−0.07)) and TV time (B = −0.25 (95%CI = −0.48;−0.03)) at the end of those four years. To conclude, in healthy older individuals, higher levels of objective SB are related to cognitive decline across a four-year follow-up, although the magnitude and clinical relevance are questionable. As preceding cognitive decline is associated with more SB across follow-up, this suggests that a bidirectional association is plausible.

## 1 Introduction

Evidence suggests that physical activity (PA) promotes healthy brain ageing, making exercise a prominent focus of dementia prevention strategies (Brini et al., 2018). Some evidence indicates that sedentary behaviour (SB; low-intensity activities with a Metabolic Equivalent of Task (MET) of < 1.5) (Gibbs et al., 2015), independent of physical activity, might also be negatively affecting cognitive health (Falck et al., 2017). Reducing SB instead of increasing exercise might be a more feasible target in older adults due to the high prevalence of SB (on average 9.2 h of SB a day) (Wheeler et al., 2017). However, current literature shows conflicting results. Bakrania et al. found associations between some specific subjective SBs and cognitive decline (Bakrania et al., 2018). Additionally, using subjective sedentary measures modest associations between SB and cognitive decline were identified in TILDA (Olanrewaju et al., 2020). In contrast, no cross-sectional associations with objectively measured SB (Wu et al., 2020), nor prospective associations with global cognitive decline (Maasakkers et al., 2020), were found in two other studies. Therefore, a recent systematic review examining the association between SB and cognition stated that evidence remains inconclusive (Olanrewaju et al., 2020). Before SB can be identified as a potential target for dementia prevention, these contradictory results need to be elucidated.

The way of measuring both the exposure SB and outcome cognition might explain part of the difference in results. Previous studies typically examined SB using single-item questionnaires, even though it is known that there are problems with the validity of such questionnaires (Healy et al., 2011). Particularly in the older population, recall bias and misinterpretation of the question causes these subjective assessments of SB to be less reliable (Herbolsheimer et al., 2018, Van Uffelen et al., 2011). Therefore, it is important to investigate the association with more reliable, objective measures. In this study we therefore look at SB measured with the GENEActiv accelerometer. To simultaneously study if this difference of objective versus subjective assessment of SB explains part of the discrepancies present in literature, we have also included the commonly used subjective measure of television time as a comparison.

Similarly, a large variety of cognitive measures have been used in previous literature, ranging in sensitivity and in the domains assessed. It is possible that the effect SB has on the brain in a healthy population, might be too subtle to be detected by less sensitive global cognitive measures such as the frequently used mini-mental state examination (MMSE) (Mazzoni et al., 1992). This motivates the use of more sensitive, domain-specific measures of cognitive function (rather than MMSE alone).

Another limitation of previous work relates to the study designs adopted to detect the possible causal link between SB and cognitive decline. Since adults with probable MCI have been shown to be more sedentary compared to their healthy cognitive peers (Falck et al., 2017), and evidence also suggests that people with dementia have higher levels of SB (Hartman et al., 2018), preclinical cognitive decline could be a cause of higher SB levels rather than SB leading to cognitive decline. To understand the complex, potential bidirectional, relation between SB and cognition, as well as remedy some of the limitations of previous studies, we approached this association by looking at it from different angles using The Irish Longitudinal Study on Ageing (TILDA). This dataset allowed us to evaluate how subjective TV time and objectively-measured SB are associated with each other, but also how they are cross-sectionally and longitudinally (four-year follow-up period) associated with global and domain-specific cognitive outcomes in healthy older adults. Secondly, by examining the reverse association between cognitive decline and future SB, we investigated the bidirectionality of the relation between SB and cognition.

## 2 Methods

### 2.1 Dataset and participants

**Fig. 1:**
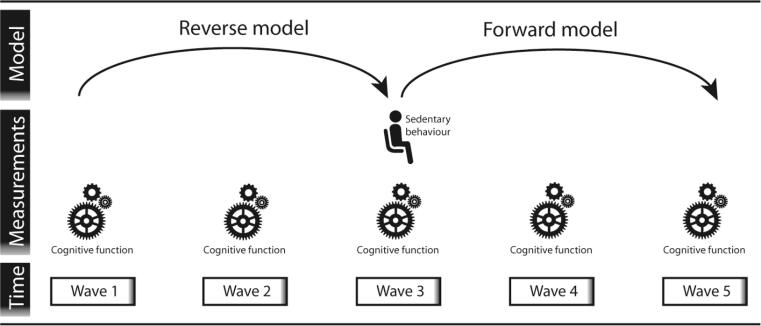
Study design showing the waves and measurements used for both the reverse and forward model, as a visual representation of both statistical models. The reverse model used change in cognitive functioning, measured between W1 and W3 as exposure variable with SB as an outcome variable. The forward model used SB at W3 as the exposure variable and cognitive functioning over W3-W5 as outcome variables.

For this investigation we used data from TILDA, a large-scale, nationally representative, longitudinal study on ageing in Ireland. Ethical approval for TILDA was received from the Health Sciences Research Ethics Committee at Trinity College Dublin, Dublin, Ireland, for additional details see (Kenny et al., 2010). Eligible participants were community-dwelling adults aged 50 years and over and their spouses (excluded from this study if < 50 years) who were able to provide informed consent. Detailed information on the cohort can be found in previous literature (Kenny et al., 2010, Kearney et al., 2011, Whelan and Savva, 2013). Different modes of data collection were used. During a computer-aided personal interview (CAPI) questions on socio-demographics, lifestyle, and physical health were asked. In the self-completion questionnaires (SCQ) more delicate topics, such as mental health, were assessed. Lastly, a comprehensive, nurse-administered health assessment was used to collect data on a wide range of objective indicators of health, physical, and mental function. Multiple waves of data collection are present in TILDA, with the first wave (W1) of data with 8,504 participants collected from 2009 to 2011 and subsequent waves collected at two-year intervals (Donoghue et al., 2018). In wave 3 (W3, collected from 2014 to 2015) SB was measured objectively with GENEActiv accelerometers in 1,596 participants. Therefore, our forward model included W3 as a baseline measurement with SB predicting cognitive functioning over W3, W4, and W5 (see Fig. 1). Our reverse model used change in cognition as measured over W1, W2, and W3 as predictor of SB at W3.

### 2.2 Sedentary behaviour, cognition, and covariates

Activity patterns were objectively measured during seven full consecutive days by the wrist worn triaxial accelerometer GENEActiv (Gravity Estimator of Normal Everyday Activity, ActivInsights Ltd, Cambridgeshire, UK). A sampling frequency of 100 Hz was used and data was converted to 15-sec epochs. Sleep time, identified with a validated algorithm (Scarlett et al., 2020), was excluded. SB was extracted from the remaining data via a modified version of the Sedentary Sphere, in which posture is determined based on arm elevation and activity intensity (Rowlands et al., 2014, Rowlands et al., 2015). SB per day was categorised based on horizontal arm elevation and low activity intensity, and averaged over the whole wear period. Subjective TV time, a frequently used surrogate measure of SB, and used primarily as a comparison variable in this study, was collected at W3 with the following question during the CAPI: “On a typical weekday, how many hours would you spend watching TV?”.

Cognitive function was operationalised in a global as well as domain-specific way. For assessment of global cognitive functioning the MMSE was used, which assesses several domains with a maximum score of 30, administered during the CAPI at all waves (Folstein et al., 1975). The domain-specific outcome measures included performance on a 10-word immediate and delayed recall task, which used one of four randomly selected word lists validated by the Health and Retirement Study (Shih et al., 2011). Immediate recall was assessed twice, where respondents heard the word list immediately before both attempts, leading to a maximum score of 20. After a short delay, respondents were asked to recall the 10 words again without hearing the list (delayed recall score). Lastly, semantic fluency was assessed by asking respondents to name as many animals as possible in one minute. The animal naming score is the total number of animal names mentioned.

The following covariates were used in order to control for potential confounding: age, sex, education, depression, body mass index (BMI), morbidity count, smoking, alcohol consumption, subjective sleep quality (e.g. trouble falling asleep), systolic and diastolic blood pressure (SBP, DBP), perceived health status, marital status, mobility limitations, and moderate-to-vigorous physical activity (MVPA). These covariates were used based on model-specific baseline measurements.

### 2.3 Statistical analysis

Pearson and Spearman correlation coefficients were calculated respectively for the subjective TV time and objective SB measures and the covariates. A mixed model analysis was used to assess the cross-sectional, forward, and reverse associations of SB and cognitive functioning. The combined cross-sectional and forward analysis consisted of a model with SB at W3 as an exposure variable and cognitive functioning as an outcome over time (W3-W5). Both a random intercept and random slope were included, with SB representing the cross-sectional association, and SB*time (follow-up in years) representing the longitudinal effect. Secondary analysis entailed stratification based on employment status. Additional secondary analysis looked at the longitudinal effect of objective SB in a non-linear way as previous literature indicated effects of total SB only to become apparent after eight hours of SB (Patterson et al., 2018, Ekelund et al., 2019). Zero to eight hours of SB was therefore used as a reference category in this analysis, with each additional hour above eight hours of SB being categorised into the next category.

The reverse model comprised a model with cognitive decline (individual linear slopes of W1-W3), corrected for baseline cognitive functioning (W1), as exposure variables and SB as an outcome variable (W3). Separate models were used for TV time and objective SB, as well as for the different cognitive outcome measures. Unadjusted, minimally adjusted (age, sex, education) and fully adjusted models were reported. Dependencies within households were controlled for in both the forward and reverse model, but not the forward models stratified by employment status due to limited number of households per subgroup. All analyses were performed with Stata 15.0 (StataCorp. 2017. Stata Statistical Software: Release 2015. College Station, TX: StataCorp LLC), with two-sided testing, and P values <0.05 considered significant.

## 3 Results

In total 1,276 participants who wore an accelerometer had valid objective activity data for the analysis (Supplementary Fig. 1). Participants who wore an accelerometer were slightly older and less likely to be employed compared to the remainder of the sample (Table 1). Over the whole eight years (W1-W5) people declined on average −0.05 (−0.07;−0.03) on the immediate recall task, −0.05 (−0.06;−0.03) on the delayed recall task, −0.00 (−0.01;0.01) on the MMSE, and −0.37 (−0.41;−0.32) on the animal naming task per year.

**Table 1:** Participant characteristics at baseline Wave 3 for the current GENEActiv sample and remainder of the whole TILDA sample.

Variable	Mean (SD) / % (n)	N	Mean (SD) / % (n)	N
	GENEActiv sample		Remaining sample	
Age (years (SD))	67.3 (9.0)	1276	66.5 (9.8)	5411
(years, range)	50 – 92		34 – 98	
Sex (% female (n))	53% (680)	1276	57% (3066)	5411
BMI (kg/m^2^ (SD))	28.7 (5.3)	1271	28.6 (5.2)	4077
SBP/DBP (mmHg (SD))	136 (19) / 81 (11)	1272	134 (20) / 81 (11)	4104
Smoker (% (n))		1276		5405
Never	45% (571)		46% (2465)	
Past	44% (567)		41% (2222)	
Current	11% (138)		13% (718)	
Employment status (% (n))		1273		5403
Employed	27% (347)		34% (1833)	
Retired	53% (676)		44% (2361)	
Other	20% (250)		22% (1209)	
Self-reported morbidities (% (n))		1276		5411
Hypertension	38% (487)		36% (1952)	
Abnormal heart rhythm	9% (117)		9% (473)	
Hyper cholesterolemia	36% (459)		36% (1944)	
Diabetes	8% (105)		9% (487)	
Self-reported TV time (hours/day (SD))	2.9 (2.0)	1276	2.8 (1.8)	5385
Objective activity pattern		1276	N.A.	
Sedentary time (hours/day (SD))	8.2 (1.8)			
MVPA time (hours/day (SD))	1.5 (0.9)			
Sleep time (hours/day (SD))	7.7 (1.2)			
Cognitive measures				
Immediate recall (count/20 (SD))	13.9 (3.0)	1276	13.6 (3.4)	5280
Delayed recall (count/10 (SD))	6.1 (2.4)	1276	6.0 (2.6)	5276
MMSE (score/30 (SD))	28.7 (1.7)	1275	28.4 (2.2)	5285
Animal naming test (count (SD))	19.2 (5.7)	1276	18.9 (6.0)	5284

Abbreviations: BMI = body mass index, SBP = systolic blood pressure, DBP = diastolic blood pressure, MVPA = moderate-to-vigorous physical activity, MMSE = mini-mental state examination.

TV time and objective SB were weakly correlated (r_s_ = 0.17, p < 0.001). Correlations were observed between both TV time and objective SB with higher age (r_s_ = 0.19 and r_s_ = 0.32), higher systolic blood pressure (r_s_ = 0.07 and r_s_ = 0.10), a higher cardiovascular morbidity count (r_s_ = 0.14 and r_s_ = 0.13), worse sleep quality (r_s_ = −0.06 and r_s_ = −0.05), and lower moderate-to-vigorous PA (r_s_ = −0.18 and r_s_ = −0.52). Furthermore, higher TV time, but not objective SB, was associated with a higher BMI (r_s_ = 0.10), depressive symptoms (r_s_ = 0.07), and worse perceived health (r_s_ = 0.14). Higher levels of SB were seen for people who were retired compared to people who were employed (8.5 vs 7.6 h of objective SB/day (p < 0.001) and 3.1 vs 2.2 vs hours of self-reported TV time/day (p < 0.001)). Supplementary Table 1 shows that, in contrast to the subjective TV time, higher levels of objective SB were associated with a higher education, within the employed group (p = 0.01). People with a higher National-Adult Reading Test (NART) score, a proxy for pre-morbid intelligence, had higher levels of objective SB (8.4 vs 7.9, p < 0.001), but lower levels of TV time (2.7 vs 3.1, p < 0.001). Distinct associations between subjective TV time and objective SB were seen for the participation in multiple social activities as well (Supplementary Table 2).

**Fig. 2:**
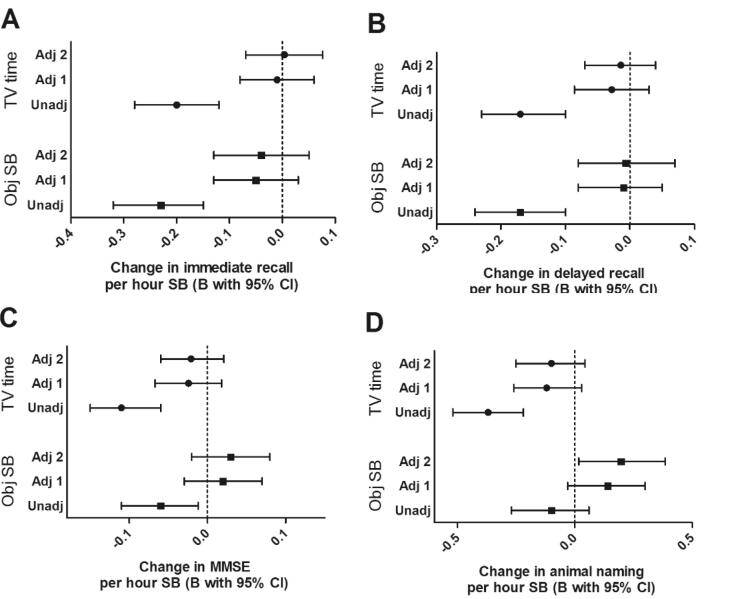
Cross-sectional unstandardized regression estimates representing the effect of one hour subjective TV time or objective sedentary behaviour on four different cognitive outcome scores with and without adjustment for potential confounders. Values shown are cross-sectional unstandardized regression estimates with 95% confidence intervals of TV time (top three) or objective SB (bottom three) in hours per day predicting cognitive function measured by four different cognitive tests. A = immediate recall, B = delayed recall, C = MMSE, D = animal naming. Three models per sedentary measure are shown where Unadj = unadjusted (n = 1276), Adj 1 = adjusted for age, sex, and education (n = 1276), Adj 2 = adjusted for age, sex, education, marital status, depression, mobility limitations, smoking, BMI, morbidities, perceived health status, systolic and diastolic blood pressure, sleep quality, alcohol consumption, and MVPA (n = 1258).

Cross-sectional analysis showed that higher SB levels were associated with lower performance on most cognitive measures (Fig. 2). Adjusting for age, sex, and education, however shifted these estimates closer to the null (in most cases) and resulted in a loss of statistical significance. After full adjustment, positive confounding was seen with the animal naming test leading to a positive association with objective SB. Stratification by employment status showed that for the immediate and delayed recall the unadjusted negative association only remained in the ‘retired’ and ‘other’ group, not in the ‘employed’ group (Supplementary Table 3). Additionally, a positive cross-sectional association between objective SB and the MMSE was found in the employed group only.

**Fig. 3:**
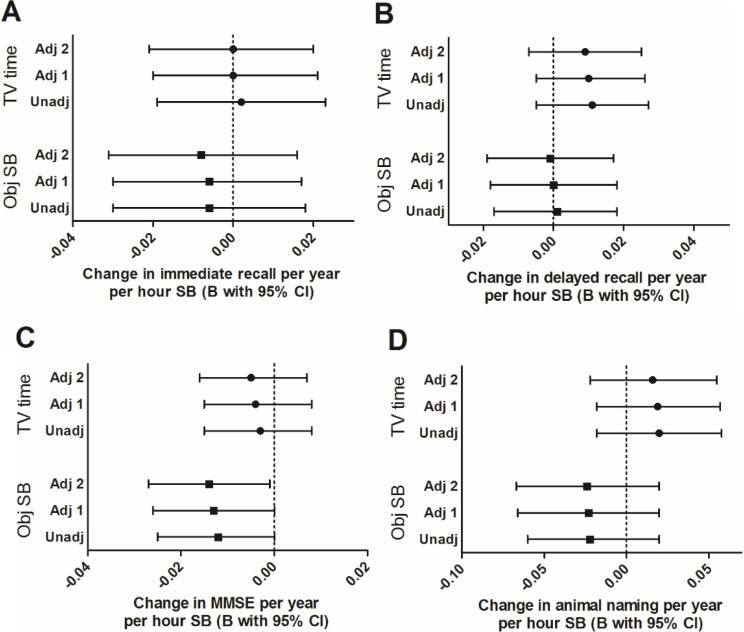
Longitudinal unstandardized regression estimates representing the effect of one hour subjective TV time or objective sedentary behaviour on four different cognitive outcome scores per year with and without adjustment for potential confounders. Values shown are longitudinal unstandardized regression estimates with 95% confidence intervals of TV time (top three) or objective SB (bottom three) in hours per day*time in years predicting cognitive function over time measured by four different cognitive tests. A = immediate recall, B = delayed recall, C = MMSE, D = animal naming. Three models per sedentary measure are shown where Unadj = unadjusted (n = 1276), Adj 1 = adjusted for age, sex, and education (n = 1276), Adj 2 = adjusted for age, sex, education, marital status, depression, mobility limitations, smoking, BMI, morbidities, perceived health status, systolic and diastolic blood pressure, sleep quality, alcohol consumption, and MVPA (n = 1258).

Longitudinally, every hour of objective SB was associated with a 0.01 (95%CI = −0.03;−0.00) decline on the MMSE per year in the fully adjusted model (Fig. 3C). No associations were found for the immediate and delayed recall or animal naming task. No effect of subjective TV time on cognition over time was seen either. The secondary analysis, looking at the effect of each additional hour above the reference eight hours of objective SB a day showed no association with both recall tasks, but a trend towards a negative association with the MMSE and animal naming task (Supplementary Fig. 2).

**Fig. 4:**
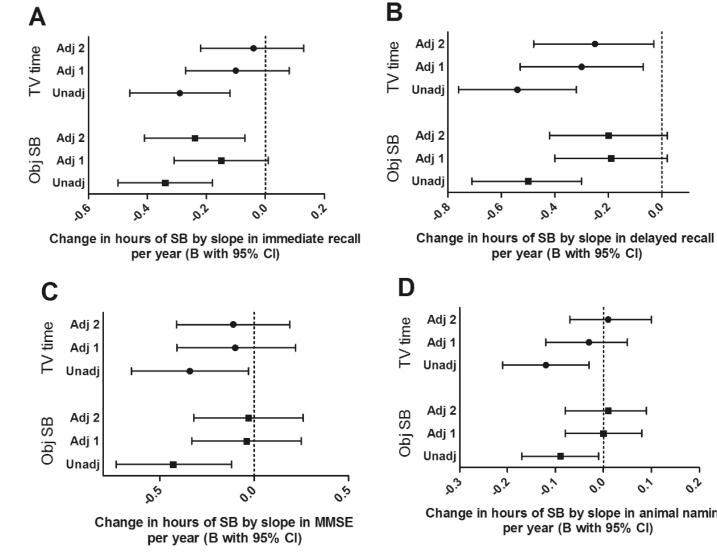
Unstandardized regression estimates representing the effect of one point of cognitive change per year of four different outcome measures on subjective TV time and objective sedentary behaviour from the reverse model with and without adjustment for potential confounders. Values shown are unstandardized regression estimates with 95% confidence intervals from the reverse models of cognitive function measured by four different cognitive tests per year predicting subjective TV time (top three) or objective SB (bottom three) in hours per day. A = immediate recall, B = delayed recall, C = MMSE, D = animal naming. Note that a decline in cognition is represented by a negative slope, multiplying that with a negative B results in an increasing effect on SB. Three models per sedentary measure are shown where Unadj = only adjusted for baseline cognitive function (n = 1196/1183/1084/1194), Adj 1 = adjusted for baseline cognitive function, age, sex, and education (n = 1196/1183/1084/1194), Adj 2 = adjusted for baseline cognitive function, age, sex, education, marital status, depression, mobility limitations, smoking, BMI, morbidities, perceived health status, systolic and diastolic blood pressure, sleep quality, alcohol consumption, and MVPA (n = 1058/1047/1057/1056).

The reverse analysis investigated if cognitive decline over the preceding four years was associated with SB at the end of those four years. The results (Fig. 4) of the unadjusted models showed that greater cognitive decline preceding the SB measurement at W3 was associated with slightly elevated levels of objective and subjective SB. In the adjusted models, where we correct for age, sex, and education, these preceding changes in MMSE and animal naming task scores were no longer significantly related to higher levels of objective nor subjective SB. However, more decline in immediate recall remained associated with higher levels of objective SB. Similarly, greater decline in delayed recall was associated with more subjective TV time. This means people who declined more on the delayed recall task during the four preceding years, were watching more TV at the end of those four years.

## 4 Discussion

This study enabled us to compare the association of subjective (i.e. TV time) and objective SB measures (i.e. accelerometery) with multiple cognitive outcomes, but also to investigate the direction of this association, in a large group of healthy older adults. First, we found that subjective TV time and objective SB measures were only weakly correlated with each other, indicating that they are non-interchangeable. Second, the negative cross-sectional associations that were found between subjective TV time and objective SB with cognition were fully explained by confounders. Third, after adjusting for confounders in our forward model, we found a small but significant longitudinal association between objective SB and decline in global cognition. No longitudinal associations were found for subjective TV time and cognitive decline. Finally, in our reverse analysis we found that faster decline in immediate and delayed recall from preceding waves 1–3 was related to elevated levels of objective SB and subjective TV time, respectively, at W3, supporting the presence of reverse causality.

Our study confirms previous work suggesting that TV time is poorly correlated with objective measures of overall SB (Stamatakis et al., 2019). Besides the accepted validity issues of subjective assessments of SB (Chastin et al., 2014), our correlation analysis also supports the hypothesis that specific types of SB might be differently distributed across the population and have different associations with demographic and socioeconomic characteristics. The increase in objective SB but reduction in subjective TV time with education among employed participants, is a clear example of that. As a consequence, different types of SB could be associated (by distinct confounding or mediation) with different health outcomes (Rhodes et al., 2012). For example, social interactions or mental engagement during sitting could partly buffer some of the negative physiological effects of being sedentary. This highlights that the context and type of SB may be relevant. This is especially important when studying a group of older adults that is partly employed and partly retired, as types of SB differ in important ways between the working population and group of retired individuals. The results stratified by employment status supported this, showing e.g., a positive cross-sectional association between objective SB and the MMSE in the employed group, probably representing higher occupational complexity. Subjective TV time and objective SB measures should therefore not be interchangeably used as markers for sitting.

In contrast with previous research (Maasakkers et al., 2020), we found that higher levels of objective SB were associated with global cognitive decline (using the MMSE). This therefore does not support our hypothesis that the sensitivity in the outcome measure is per se responsible for the null findings of previous research. Though, as the effect size of the association between SB and MMSE (a decrease of only 0.01 in MMSE per year for every hour of SB) was very small, it is not clinically meaningful. Nevertheless, in our secondary analysis a trend towards a very small association between additional hours of objective SB (after eight hours of SB) and greater decline in verbal fluency (animal naming test) over time was also observed. Previous literature has shown that SB is related to detrimental cardiovascular health effects (Carter et al., 2017). In turn, these have been related to increased risk of dementia (Whitmer et al., 2005). We therefore hypothesise that, via this pathway, sitting could have a negative effect on cognitive health, but due to the indirect effect it is likely to be characterised by a large lag. SB does not directly cause hypertension or reduced glycemic control, nor do these effects immediately lead to brain damage that underlies cognitive decline. This suggests that the cognitive effects of excessive SB might only become apparent after many years, and may therefore not be strongly evident during the four year period studied here. This is especially true in a population of healthy older adults with minimal cognitive decline. Consequently, primary prevention strategies need to take into account that lowering SB may need to be successful over longer periods of time to gain potential benefit.

A decline in the immediate and delayed recall tasks was shown to be associated with slightly higher levels of objective SB and subjective TV time, respectively, at W3. As we have no records of how much people were sitting at W1, it is possible that this association is due to reverse causation itself. However, combining this information with previous literature makes it plausible that a reverse association might be present between these two factors. Decades before a clinical manifestation of neurodegenerative diseases is seen, and even in the presence of only slight cognitive decline, neuropathological changes may already be evident (Daulatzai, 2017). These neurological changes are known to be accompanied by apathy and loss of initiative, even in the first stages of neurodegenerative diseases (Robert et al., 2009). This could be another pathway through which cognitive decline is associated with SB. In contrast to the forward association that might need a longer timespan to become visible, this reverse association could become evident within a shorter time period if slight indications of cognitive decline are present. Therefore, it is necessary to take the possibility of this reverse association into account, both when interpreting SB as an etiological factor in health outcomes as well as in designing interventions that aim to reduce SB.

A few limitations have to be kept in mind. First of all, the GENEActiv device that was used to assess objective total SB has some downfalls due to the fact that it is a wrist-worn accelerometer. Despite the fact that SB was characterised in a very rigorous way, e.g., not only based on activity intensity but also based on the posture of the hand, some error of the actual hours of sitting per day might be present. A thigh-worn accelerometer that can differentiate based on body posture, is therefore the superior measurement technique. Additionally, no cognitive tests more sensitive to vascular cognitive impairments, like executive functioning tests, were used. Moreover, despite the design of the study including both reverse and forward models, no causal claims can be made based on these results. Lastly, the subsample of TILDA participants that was included in this analysis was not fully representative of the whole sample. Therefore, generalising these results to a wider population should be done with caution, taking into account the different distribution of specific sedentary types in a potential younger and more likely to be employed population.

## 5 Conclusion

How SB is measured, e.g., by subjective TV time or objective total sedentary time, may explain the contradictory findings of studies relating SB to health outcomes. As sedentary behaviour is so strongly related to many aspects of life, confounding may play an important role when SB and health outcomes appear to be related. The underlying factors for null findings in research on SB and cognition, are however, besides the measurement techniques used, probably more related to the time period under investigation. Our data, combined with previous literature, provide some support for a link, albeit weak, between SB and cognitive decline in healthy older individuals. This association, however, potentially needs a longer time period than four years to more strongly become visible. Additionally, preceding decline in memory function was associated with increased SB, creating a need to be cautious for reverse causation when researching health effects of SB. Together, this could make a bidirectional association between objective SB and cognitive function plausible.

## 6 Ethics approval and consent to participate

Ethical approval for TILDA was received from the Health Sciences Research Ethics Committee at Trinity College Dublin, Dublin, Ireland, for additional details see Kenny et al. (Kenny et al., 2010). All participants gave written informed consent.

## 7 Availability of data and materials

Researchers interested in using TILDA data may access the data for free from the following site of the Irish Social Science Data Archive at University College Dublin (www.ucd.ie/issda/data/tilda/).

## 8 Contributions

CM, JC, DT, RAK, JF, and RM designed the study. SS, RAK, and JF were involved in data acquisition. CM, SS, JF and RM performed the data analysis and interpretation of data. JC, DT, and RAK provided critical feedback for data analysis. CM, JF, and RM prepared figures and wrote the manuscript. JC, SS, DT, and RAK critically revised the manuscript. All authors read and approved the final manuscript.

## Funding

TILDA is funded by the Department of Health, Irish Life, and The Atlantic Philanthropies. CM was supported by a personal international research internship grant awarded by Alzheimer Nederland.

Sponsors played no role in the design, execution, analysis and interpretation of data, or writing of the paper.

## Declaration of Competing Interest

The authors declare that they have no known competing financial interests or personal relationships that could have appeared to influence the work reported in this paper.
