# Supplementary material for: Is there a bidirectional association between sedentary behaviour and cognitive decline in older adults? Findings from the Irish Longitudinal Study on Ageing
Source: Prev Med Rep. 2021 Jul 1;23:101423. doi: 10.1016/j.pmedr.2021.101423 (PMC8259404; doi:10.1016/j.pmedr.2021.101423)
Supplement: Supplementary data 1 [file mmc1.docx]

**Supplementary information**

Index

Supplementary Figure 1 – Flow chart of whole wave 3 TILDA sample to current GENEActiv sample.

Supplementary Table 1 – Sedentary times (hours/day) broken down for employment status and education level.

Supplementary Table 2 – Associations between subjective TV time and objective sedentary behaviour with participation in social activities.

Supplementary Table 3 – Unstandardised regression estimates of the forward analysis expressing the effect of sedentary behavior on cognitive function measured by four different measures stratified by employment status.

Supplementary Figure 2 – Secondary longitudinal unstandardized regression estimates representing the effect of one additional hour objective sedentary behaviour after eight hours of sedentary behaviour a day on four different cognitive outcome scores per year with and without adjustment for potential confounders.

**Supplementary Figure 1 – Flow chart of whole wave 3 TILDA sample to current GENEActiv sample.**

**Supplementary Table 1 – Sedentary times (hours/day) broken down for employment status and education level.**

|  | **Employed** | | **Retired** | | **Other** | |
| --- | --- | --- | --- | --- | --- | --- |
| **Education** | **Subj. TV** | **Obj. total SB** | **Subj. TV** | **Obj. total SB** | **Subj. TV** | **Obj. total SB** |
| **Primary** | 2.9 (1.4) | 7.1 (1.5) | 3.7 (2.9) | 8.6 (1.9) | 4.0 (2.9) | 8.4 (1.8) |
| **Secondary** | 2.2 (1.2) | 7.5 (1.7) | 3.2 (1.7) | 8.4 (1.9) | 2.9 (1.8) | 7.8 (1.9) |
| **Third** | 2.0 (1.8) | 7.8 (1.6) | 2.5 (1.5) | 8.6 (1.7) | 2.8 (1.6) | 8.0 (1.8) |

*Values shown are means (SD). Subj=subjective assessment, Obj=objective accelerometer assessment. n=347 employed, 676 retired, 250 other.*

**Supplementary Table 2 – Associations between subjective TV time and objective sedentary behaviour with participation in social activities.**

|  | **Objective SB** | |  | **TV time** | |
| --- | --- | --- | --- | --- | --- |
|  | **r_s_** | **P-value** |  | **r_s_** | **P-value** |
| **Watch TV** | 0.04 | 0.22 |  | 0.32 | <0.001 |
| **Go out to films, plays and concerts** | 0.01 | 0.85 |  | -0.11 | <0.001 |
| **Attend classes and lectures** | 0.02 | 0.46 |  | -0.17 | <0.001 |
| **Travel for pleasure** | -0.00 | 0.98 |  | -0.07 | 0.02 |
| **Work in garden home or car** | -0.05 | 0.13 |  | -0.09 | 0.001 |
| **Read books or magazines** | 0.09 | 0.003 |  | -0.07 | 0.03 |
| **Listen to music/radio** | -0.03 | 0.25 |  | -0.09 | 0.003 |
| **Spend time on hobbies or creative** | 0.04 | 0.18 |  | -0.15 | <0.001 |
| **Play cards, bingo games** | 0.06 | 0.06 |  | 0.01 | 0.86 |
| **Go to pub** | -0.13 | <0.001 |  | -0.02 | 0.53 |
| **Eat out of house** | 0.06 | 0.03 |  | -0.08 | 0.006 |
| **Participate in sport activities or exercise** | -0.06 | 0.03 |  | -0.15 | <0.001 |
| **Visit to or from family or friends** | 0.05 | 0.10 |  | -0.02 | 0.53 |
| **Do voluntary work** | -0.02 | 0.57 |  | -0.20 | <0.001 |

*Values shown are Spearman correlations with corresponding p-values. Positive correlations represent an association between higher levels of sedentary behaviour and a higher likeliness to participate in the particular social activity.*

**Supplementary Table 3 – Unstandardised regression estimates of the forward analysis expressing the effect of sedentary behavior on cognitive function measured by four different measures stratified by employment status.**

Supplementary Table 3.1 – Unstandardised regression estimates of objective sedentary behavior predicting immediate recall scores.

|  | **Cross-sectional estimate** | | | **Slope estimate** | | |
| --- | --- | --- | --- | --- | --- | --- |
|  | **Employed** | **Retired** | **Other** | **Employed** | **Retired** | **Other** |
| **Unadjusted** | 0.02 (-0.15 ; 0.19) | -0.23 (-0.35 ; -0.10) | -0.25 (-0.44 ; -0.06) | 0.01 (-0.04 ; 0.06) | 0.00 (-0.03 ; 0.03) | -0.02 (-0.08 ; 0.03) |
| **Minimally adjusted** | 0.03 (-0.13 ; 0.20) | -0.06 (-0.17 ; 0.05) | -0.10 (-0.28 ; 0.07) | 0.01 (-0.04 ; 0.06) | 0.00 (-0.03 ; 0.03) | -0.02 (-0.08 ; 0.03) |
| **Fully adjusted** | 0.03 (-0.16 ; 0.21) | -0.06 (-0.18 ; 0.07) | -0.11 (-0.30 ; 0.09) | 0.01 (-0.04 ; 0.06) | -0.00 (-0.03 ; 0.03) | -0.03 (-0.09 ; 0.03) |

Supplementary Table 3.2 – Unstandardised regression estimates of subjective TV time predicting immediate recall scores.

|  | **Cross-sectional estimate** | | | **Slope estimate** | | |
| --- | --- | --- | --- | --- | --- | --- |
|  | **Employed** | **Retired** | **Other** | **Employed** | **Retired** | **Other** |
| **Unadjusted** | -0.12 (-0.30 ; 0.06) | -0.17 (-0.28 ; -0.06) | -0.16 (-0.32 ; -0.00) | -0.01 (-0.06 ; 0.04) | 0.01 (-0.02 ; 0.04) | 0.00 (-0.04 ; 0.05) |
| **Minimally adjusted** | 0.03 (-0.14 ; 0.20) | -0.01 (-0.11 ; 0.08) | -0.02 (-0.17 ; 0.13) | -0.02 (-0.07 ; 0.04) | 0.01 (-0.02 ; 0.04) | 0.00 (-0.04 ; 0.05) |
| **Fully adjusted** | 0.03 (-0.14 ; 0.20) | -0.00 (-0.10 ; 0.10) | -0.02 (-0.17 ; 0.14) | -0.02 (-0.07 ; 0.03) | 0.01 (-0.02 ; 0.04) | 0.00 (-0.04 ; 0.05) |

*Estimates are shown with 95% confidence intervals. Employed n=347, retired n=676, other n=250. Minimally adjusted model is corrected for age, sex, and education. Fully adjusted model furthermore corrects for marital status, depression, smoking, BMI, morbidities, MVPA, SBP, DPB, sleep quality, and alcohol consumption.*

Supplementary Table 3.3 – Unstandardised regression estimates of objective sedentary behavior predicting delayed recall scores.

|  | **Cross-sectional estimate** | | | **Slope estimate** | | |
| --- | --- | --- | --- | --- | --- | --- |
|  | **Employed** | **Retired** | **Other** | **Employed** | **Retired** | **Other** |
| **Unadjusted** | 0.06 (-0.09 ; 0.20) | -0.16 (-0.26 ; -0.06) | -0.18 (-0.33 ; -0.02) | -0.01 (-0.05 ; 0.02) | 0.00 (-0.02 ; 0.03) | 0.02 (-0.02 ; 0.06) |
| **Minimally adjusted** | 0.06 (-0.07 ; 0.20) | -0.02 (-0.11 ; 0.07) | -0.05 (-0.20 ; 0.09) | -0.01 (-0.05 ; 0.02) | 0.00 (-0.02 ; 0.03) | 0.02 (-0.02 ; 0.06) |
| **Fully adjusted** | 0.05 (-0.11 ; 0.20) | -0.02 (-0.11 ; 0.08) | -0.04 (-0.19 ; 0.11) | -0.01 (-0.05 ; 0.02) | 0.00 (-0.02 ; 0.03) | 0.02 (-0.03 ; 0.06) |

Supplementary Table 3.4 – Unstandardised regression estimates of subjective TV time predicting delayed recall scores.

|  | **Cross-sectional estimate** | | | **Slope estimate** | | |
| --- | --- | --- | --- | --- | --- | --- |
|  | **Employed** | **Retired** | **Other** | **Employed** | **Retired** | **Other** |
| **Unadjusted** | -0.09 (-0.24 ; 0.06) | -0.13 (-0.22 ; -0.05) | -0.18 (-0.31 ; -0.05) | 0.01 (-0.03 ; 0.05) | 0.01 (-0.01 ; 0.03) | 0.03 (-0.00 ; 0.07) |
| **Minimally adjusted** | 0.04 (-0.10 ; 0.18) | -0.02 (-0.10 ; 0.05) | -0.08 (-0.20 ; 0.04) | 0.01 (-0.03 ; 0.05) | 0.01 (-0.01 ; 0.03) | 0.03 (-0.00 ; 0.07) |
| **Fully adjusted** | 0.03 (-0.11 ; 0.17) | -0.01 (-0.09 ; 0.06) | -0.08 (-0.21 ; 0.04) | 0.01 (-0.03 ; 0.04) | 0.01 (-0.02 ; 0.03) | 0.03 (-0.00 ; 0.07) |

*Estimates are shown with 95% confidence intervals. Employed n=347, retired n=676, other n=250. Minimally adjusted model is corrected for age, sex, and education. Fully adjusted model furthermore corrects for marital status, depression, smoking, BMI, morbidities, MVPA, SBP, DPB, sleep quality, and alcohol consumption.*

Supplementary Table 3.5 – Unstandardised regression estimates of objective sedentary behavior predicting MMSE scores.

|  | **Cross-sectional estimate** | | | **Slope estimate** | | |
| --- | --- | --- | --- | --- | --- | --- |
|  | **Employed** | **Retired** | **Other** | **Employed** | **Retired** | **Other** |
| **Unadjusted** | 0.11 (0.04 ; 0.18) | -0.08 (-0.16 ; -0.01) | -0.03 (-0.14 ; 0.09) | -0.03 (-0.05 ; -0.00) | -0.01 (-0.03 ; 0.01) | -0.02 (-0.05 ; 0.01) |
| **Minimally adjusted** | 0.11 (0.05 ; 0.18) | 0.00 (-0.07 ; 0.07) | 0.04 (-0.07 ; 0.15) | -0.03 (-0.05 ; -0.00) | -0.01 (-0.03 ; 0.01) | -0.02 (-0.05 ; 0.01) |
| **Fully adjusted** | 0.11 (0.03 ; 0.19) | -0.01 (-0.09 ; 0.06) | 0.10 (-0.02 ; 0.22) | -0.03 (-0.05 ; -0.00) | -0.01 (-0.03 ; 0.01) | -0.02 (-0.05 ; 0.01) |

Supplementary Table 3.6 – Unstandardised regression estimates of subjective TV time predicting MMSE scores.

|  | **Cross-sectional estimate** | | | **Slope estimate** | | |
| --- | --- | --- | --- | --- | --- | --- |
|  | **Employed** | **Retired** | **Other** | **Employed** | **Retired** | **Other** |
| **Unadjusted** | -0.11 (-0.18 ; -0.03) | -0.07 (-0.14 ; -0.01) | -0.09 (-0.19 ; 0.01) | 0.00 (-0.02 ; 0.03) | -0.01 (-0.03 ; 0.01) | 0.01 (-0.02 ; 0.03) |
| **Minimally adjusted** | -0.05 (-0.12 ; 0.02) | -0.00 (-0.06 ; 0.06) | -0.03 (-0.12 ; 0.07) | 0.00 (-0.02 ; 0.03) | -0.01 (-0.03 ; 0.01) | 0.01 (-0.02 ; 0.03) |
| **Fully adjusted** | -0.06 (-0.13 ; 0.02) | 0.00 (-0.06 ; 0.06) | -0.03 (-0.13 ; 0.07) | 0.00 (-0.02 ; 0.03) | -0.01 (-0.03 ; 0.01) | 0.01 (-0.02 ; 0.03) |

*Estimates are shown with 95% confidence intervals. Employed n=347, retired n=675, other n=250. Minimally adjusted model is corrected for age, sex, and education. Fully adjusted model furthermore corrects for marital status, depression, smoking, BMI, morbidities, MVPA, SBP, DPB, sleep quality, and alcohol consumption.*

Supplementary Table 3.7 – Unstandardised regression estimates of objective sedentary behavior predicting animal naming scores.

|  | **Cross-sectional estimate** | | | **Slope estimate** | | |
| --- | --- | --- | --- | --- | --- | --- |
|  | **Employed** | **Retired** | **Other** | **Employed** | **Retired** | **Other** |
| **Unadjusted** | 0.30 (-0.03 ; 0.64) | -0.16 (-0.40 ; 0.08) | -0.02 (-0.39 ; 0.35) | -0.03 (-0.12 ; 0.06) | -0.05 (-0.11 ; 0.01) | 0.07 (-0.02 ; 0.14) |
| **Minimally adjusted** | 0.29 (-0.04 ; 0.62) | 0.09 (-0.14 ; 0.32) | 0.22 (-0.13 ; 0.57) | -0.03 (-0.12 ; 0.06) | -0.05 (-0.11 ; 0.01) | 0.07 (-0.01 ; 0.15) |
| **Fully adjusted** | 0.29 (-0.09 ; 0.66) | 0.10 (-0.15 ; 0.35) | 0.35 (-0.03 ; 0.74) | -0.03 (-0.12 ; 0.06) | -0.05 (-0.11 ; 0.02) | 0.06 (-0.03 ; 0.14) |

Supplementary Table 3.8 – Unstandardised regression estimates of subjective TV time predicting animal naming scores.

|  | **Cross-sectional estimate** | | | **Slope estimate** | | |
| --- | --- | --- | --- | --- | --- | --- |
|  | **Employed** | **Retired** | **Other** | **Employed** | **Retired** | **Other** |
| **Unadjusted** | -0.39 (-0.75 ; -0.04) | -0.29 (-0.49 ; -0.08) | -0.37 (-0.68 ; -0.06) | 0.12 (0.03 ; 0.22) | -0.02 (-0.07 ; 0.03) | 0.06 (-0.01 ; 0.12) |
| **Minimally adjusted** | -0.27 (-0.62 ; 0.07) | -0.03 (-0.22 ; 0.17) | -0.23 (-0.53 ; 0.07) | 0.12 (0.03 ; 0.21) | -0.02 (-0.07 ; 0.03) | 0.06 (-0.01 ; 0.13) |
| **Fully adjusted** | -0.26 (-0.60 ; 0.08) | -0.02 (-0.22 ; 0.17) | -0.29 (-0.60 ; 0.02) | 0.12 (0.02 ; 0.21) | -0.02 (-0.07 ; 0.03) | 0.06 (-0.01 ; 0.12) |

*Estimates are shown with 95% confidence intervals. Employed n=347, retired n=676, other n=250. Minimally adjusted model is corrected for age, sex, and education. Fully adjusted model furthermore corrects for marital status, depression, smoking, BMI, morbidities, MVPA, SBP, DPB, sleep quality, and alcohol consumption.*

**Supplementary Figure 2 – Secondary longitudinal unstandardized regression estimates representing the effect of one additional hour objective sedentary behaviour after eight hours of sedentary behaviour a day on four different cognitive outcome scores per year with and without adjustment for potential confounders.**

*
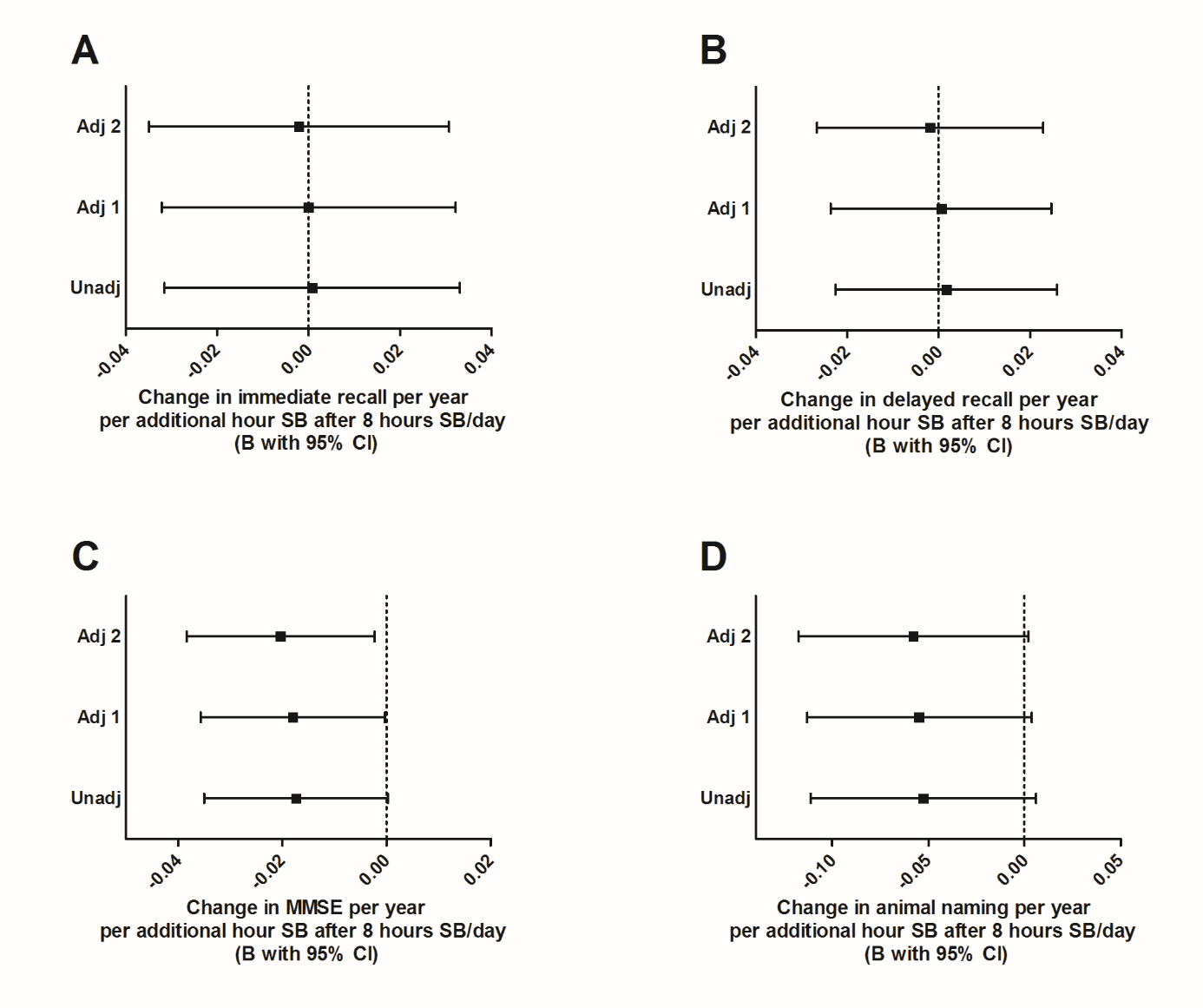
*

*Values shown are longitudinal unstandardized regression estimates with 95% confidence intervals of each additional hour of objective sedentary behaviour after eight hours of sedentary behaviour a day, in hours per day*time in years predicting cognitive function over time measured by four different cognitive tests. A=immediate recall, B=delayed recall, C=MMSE, D=animal naming. Three models per sedentary measure are shown where unadj = unadjusted (n=1276), Adj 1 = adjusted for age, sex, and education (n=1276), Adj 2 = adjusted for age, sex, education, marital status, depression, mobility limitations, smoking, BMI, morbidities, perceived health status, systolic and diastolic blood pressure, sleep quality, alcohol consumption, and MVPA (n=1258).*
